# Effect of a Cytoprotective Dose of Dehydroleucodine, Xanthatin, and 3-Benzyloxymethyl-5*H*-furan-2-one on Gastric Mucosal Lesions Induced by Mast Cell Activation

**DOI:** 10.3390/ijms22115983

**Published:** 2021-06-01

**Authors:** Mariano Ezequiel Vera, María Laura Mariani, Cristina Aguilera, Alicia Beatriz Penissi

**Affiliations:** Instituto de Histología y Embriología “Dr. Mario H. Burgos” (IHEM-CCT Mendoza-CONICET), Facultad de Ciencias Médicas, Universidad Nacional de Cuyo, Casilla de Correo 56, 5500 Mendoza, Argentina; veramarianoezequiel@yahoo.com.ar (M.E.V.); mariani.laura@gmail.com (M.L.M.); apenissi@mendoza-conicet.gob.ar (C.A.)

**Keywords:** peptic ulcer, gastric cytoprotection, mast cells, dehydroleucodine, xanthatin, 3-benzyloxymethyl-5*H*-furan-2-one

## Abstract

The aim of this study was to determine whether the lactones dehydroleucodine, xanthatin and 3-benzyloxymethyl-5H-furan-2-one, would be effective in an animal model of gastric ulcer induced by mast cell activation. Rats were divided into ten groups. Treatments were repeated for four days. The degree of gastric erosion was assessed with a scoring system and histological preparations. Gastric mast cell morphology was analyzed by histological procedures. Serum serotonin levels were determined as markers of mast cell activation. Statistical analyses were done using ANOVA and Tukey–Kramer test. We demonstrated that the repeated administration of compound 48/80 results in extensive mucosal lesions in the gastric mucosa and that such lesions occurred in association with mast cell degranulation and a significant increase of serum serotonin. We showed that these lesions were prevented by dehydroleucodine, xanthatin, and 3-benzyloxymethyl-5H-furan-2-one and that this effect was similar to that obtained with sodium cromoglycate. In conclusion, the results of the present study indicate that the optimal gastric cytoprotective dose of dehydroleucodine, xanthatin, and 3-benzyloxymethyl-5*H*-furan-2-one is efficacious in an animal model of gastric ulcer induced by mast cell activation. Our findings suggest that these lactones could be valuable tools for designing novel therapeutic agents for digestive disorders associated with inappropriate mast cell activation.

## 1. Introduction

Gastric ulcer disease is a major chronic digestive disorder affecting millions of people worldwide and being one of the leading causes of morbidity and mortality [1]. It is the result of an imbalance between endogenous protective factors of the gastric mucosa and aggressive factors [2,3]. This condition is characterized by discontinuation in the stomach mucosa lining because of gastric acid or pepsin secretion induced by different types of triggers. Although this disorder has various causes, *Helicobacter pylori* and non-steroidal anti-inflammatory drugs account for the majority of the disease etiology [4,5,6]. Gastric ulcer disease has decreased significantly but has not disappeared. Effective treatment for this condition and its complications remains a significant therapeutic challenge at present [7].

Several groups have demonstrated the importance of mast cells as key players for the regulation of the gastric mucosal barrier, both in physiological and pathological conditions [8,9,10].

Mast cells are constitutively found in the gastrointestinal tract. The three primary physiological functions of gastrointestinal mast cells comprise the regulation of gastrointestinal functions, namely epithelial and endothelial functions, crosstalk with the enteric nervous system, and contribution to the host defense against bacterial, viral and parasitic agents [11]. In addition, mast cells have been found to play an increasingly important role in the pathophysiology of gastrointestinal diseases, such as tumorigenesis and gastric ulcers [12,13,14]. Mast cells have been implicated in peptic ulcer pathogenesis in several studies [15,16,17]. These cells are involved in the pathogenesis of gastric ulcer disease with or without *Helicobacter pylori* infection. Mast cells have a critical immunoregulatory function, particularly at the mucosal border between the body and the environment. Due to the large interface of the gastrointestinal tract with the environment, mast cell overproduction or overactivation can lead to gastrointestinal disorders [10]. Mast cells are involved in gastric mucosal inflammation, mucus secretion, smooth muscle contraction, gastric acid secretion, and wound healing [18]. In the case of ulcers produced by non-steroidal anti-inflammatory drugs, these compounds induce gastric acid secretion through mast cell activation [19]. Mast cells also respond to both acute and chronic stress. The mast cell–enteric nerve association provides a physiologic means for bidirectional communication between the central nervous system and intestinal tract through which stress may influence gastrointestinal function [10]. Stressful conditions result in alterations of brain–gut interactions, leading to the development of a broad array of gastrointestinal disorders, including peptic ulcer disease [20]. This background could lead to the development of new and promising therapeutic opportunities for gastric ulcer disorders [10].

**Figure 1:**
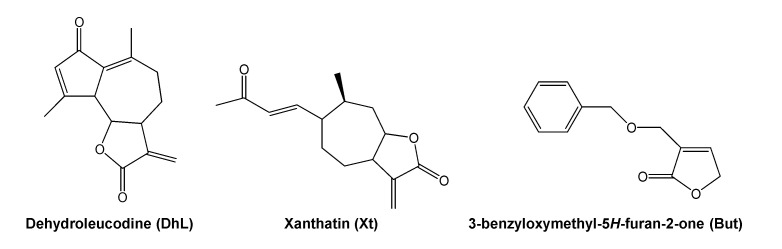
Chemical structure of dehydroleucodine, xanthatin and 3-benzyloxymethyl-5*H*-furan-2-one.

Dehydroleucodine (an α,β-unsaturated sesquiterpene lactone isolated from *Artemisia douglasiana* Besser, popularly known as “matico”), xanthatin (an α,β-unsaturated xanthanolide sesquiterpene isolated from *Xanthium cavanillesii* Schouw, popularly known as “abrojo grande”) and 3-benzyloxymethyl-5*H*-furan-2-one (a semisynthetic butenolide), prevent, in vivo, gastric and duodenal damages in response to necrosis-inducing agents in a dose-dependent manner, 40 mg/kg of body weight being the optimal gastroduodenal cytoprotective dose [21,22,23,24]. The chemical structure of dehydroleucodine, xanthatin, and 3-benzyloxymethyl-5*H*-furan-2-one can be seen in Figure 1. These lactones inhibit, in vitro, rat peritoneal and human LAD2 mast cell degranulation induced by compound 48/80, calcium ionophore A23187, and pro-inflammatory neuropeptides [25,26,27]. Based on these previous findings, we hypothesized that the lactones could act as mast cell stabilizers in the intact animal. However, this had not been tested so far.

The aim of this study was to determine whether the optimal gastric cytoprotective dose (40 mg/kg of body weight) of dehydroleucodine, xanthatin, and 3-benzyloxymethyl-5*H*-furan-2-one prevents the development of gastric lesions in an animal model of gastric ulcer induced by mast cell activation.

## 2. Results

### 2.1. Macroscopic Evaluation of Stomachs and Ulcer Index Values

**Figure 2:**
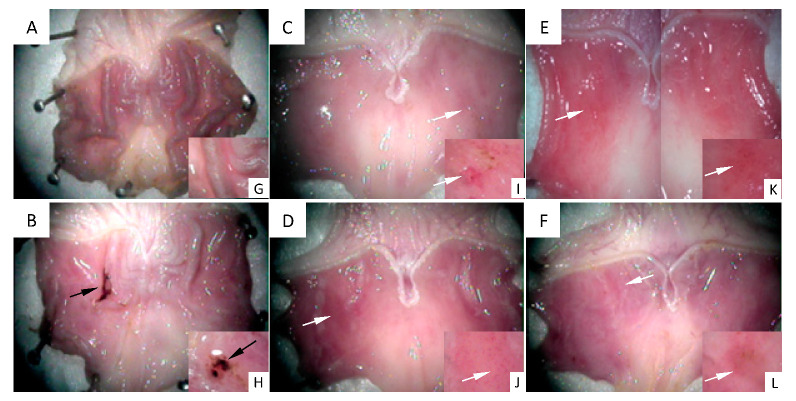
Images of the stomachs’ inner surface are seen under a stereoscopic magnifying glass. (**A**) control group ((**G**) details of gastric roughness); (**B**) 48/80 group ((**H**) details of gastric ulcer); (**C**) DhL + 48/80 group; (**D**) Xt + 48/80 group; (**E**) But + 48/80 group; (**F**) Crgl + 48/80 group ((**I**–**L**) details of mild hyperemia). Black arrows: gastric ulcer. White arrows: mild hyperemia.

Figure 2 shows images of the stomachs’ inner surface seen under a stereoscopic magnifying glass. Figure 2A shows the gastric mucosa surface representative of the control group, in which a healthy structure with typical gastric roughness can be clearly identified. Figure 2B shows the surface of a stomach representative of the 48/80 group. An ulcer perforated in the gastric mucosa, as well as mucus and hemorrhage, can be observed. Figure 2C–F shows the gastric mucosa surfaces representative of the experimental groups treated with DhL, Xt, But, or Crgl, plus compound 48/80. No gastric ulcers or injuries are seen in these groups. Regions with mild hyperemia are observed. Figure 2G–L shows details of gastric roughness (Figure 2G), gastric ulcer (Figure 2H), and mild hyperemia (Figure 2I–L).

**Figure 3:**
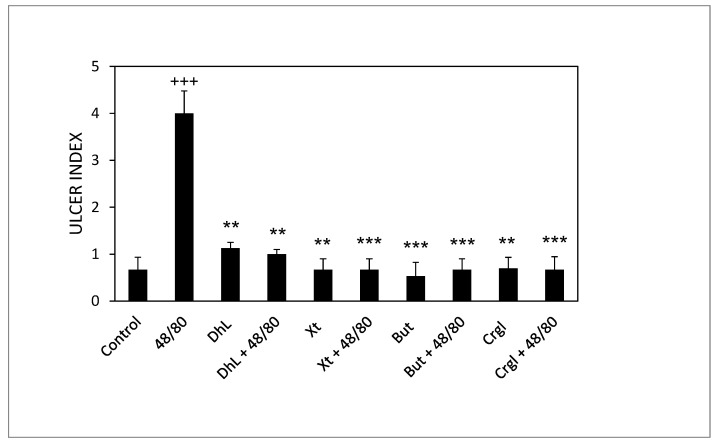
Effect of DhL, Xt, But, and Crgl on gastric mucosal damage induced by compound 48/80. Values of ulcer index are expressed as mean ± SEM +++ *p* < 0.001 when compared with the control group, ** *p* < 0.01 when compared with the compound 48/80 group, and *** *p* < 0.001 when compared with the compound 48/80 group.

Figure 3 shows the ulcerogenic index values corresponding to the different experimental groups. The ulcerogenic index of the 48/80 group’s stomachs is significantly higher than that of the control group (*p* < 0.001). Groups treated with DhL, Xt, But or Crgl, plus 48/80 compound, presented ulcerogenic indexes significantly lower than those corresponding to the 48/80 group (*p* < 0.01 for DhL and *p* < 0.001 for Xt, But, and Crgl). There were no statistically significant differences between the ulcerogenic indexes of the groups treated with the lactones and those of the control group. There were no significant differences either when comparing the ulcerogenic indexes of the lactones-treated groups among them.

### 2.2. Histological Analysis

**Figure 4:**
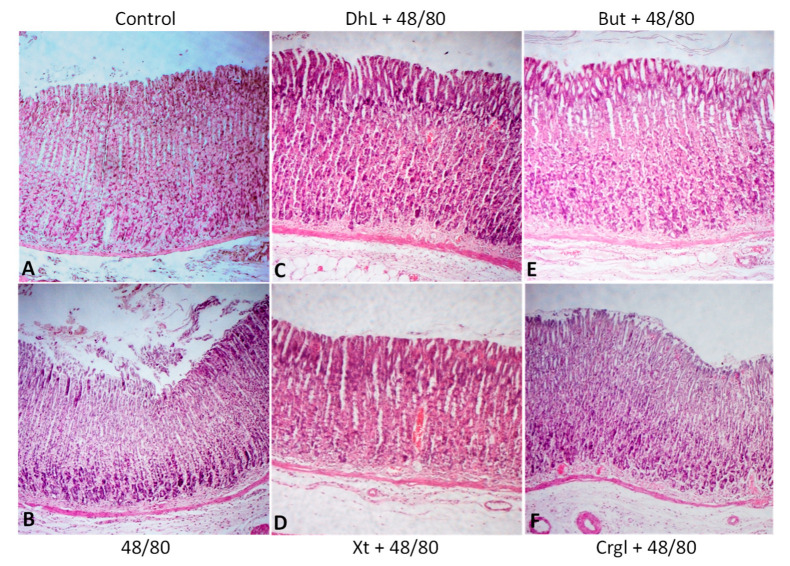
Paraffin sections (6 µm) of gastric walls stained by hematoxylin-eosin. Gastric mucosa can be seen. (**A**) Control; (**B**) compound 48/80; (**C**) DhL + compound 48/80; (**D**) Xt + compound 48/80; (**E**) But + compound 48/80; (**F**) Crgl + compound 48/80. ×100.

**Figure 5:**
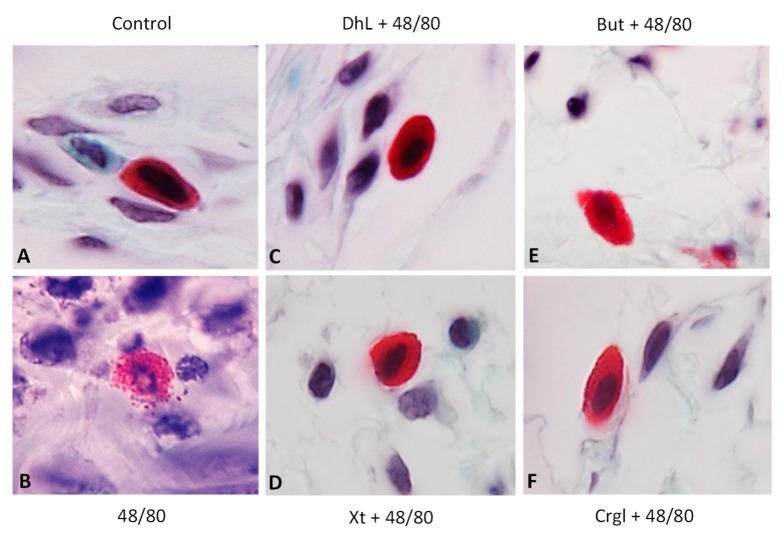
Paraffin sections (6 µm) of gastric walls stained by safranin. Representative mast cells can be seen. (**A**) Control; (**B**) compound 48/80; (**C**) DhL + compound 48/80; (**D**) Xt + compound 48/80; (**E**) But + compound 48/80; (**F**) Crgl + compound 48/80. ×600.

**Figure 6:**
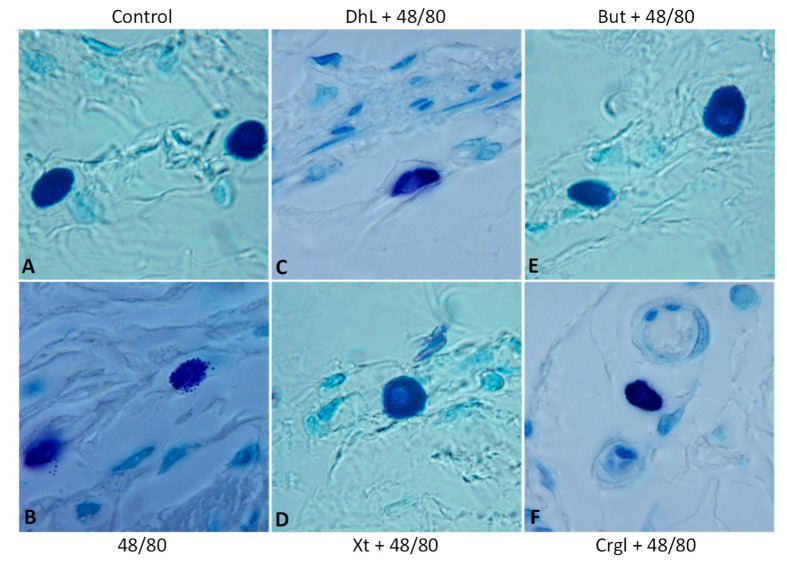
Paraffin sections (6 µm) of gastric walls stained by toluidine blue. Representative mast cells can be seen. (**A**) Control; (**B**) compound 48/80; (**C**) DhL + compound 48/80; (**D**) Xt + compound 48/80; (**E**) But + compound 48/80; (**F**) Crgl + compound 48/80. ×600.

Figure 4 shows micrographs of histological sections of hematoxylin-eosin-stained rat stomachs. In these images, it is possible to observe the histoarchitecture of the gastric mucosa representative of the different experimental groups. In Figure 5 and Figure 6, images of histological sections of rat stomachs stained with safranin and toluidine blue, respectively, are identified. Mast cells representative of the different experimental groups are shown in these micrographs.

The mucosa of the control group (Figure 4A) shows normal histological characteristics. It is possible to observe the lining epithelium, the glandular epithelium, the lamina propria, and the muscularis of the mucosa without structural alterations. Mast cells corresponding to this group (Figure 5A and Figure 6A) are pleomorphic and have abundant cytoplasmic granules. This cell population does not have morphological characteristics of degranulation.

The mucosa of animals treated with compound 48/80 (Figure 4B) shows evident injuries, mainly disruptions of the lining epithelium, as well as damage in the isthmus and in the neck of gastric glands. Blood, remnants of injured tissue, and some mucus filaments are observed in the lumen. Most of the mast cells belonging to this experimental group (Figure 5B and Figure 6B) present morphological characteristics of degranulation. Mast cell granules released from the cell can be observed.

The mucosa of animals treated with the lactones (Figure 4C–F) shows morphological characteristics similar to those of normal mucosa. Mast cells corresponding to these experimental groups (Figure 5C–F and Figure 6C–F) show morphological characteristics of stabilization, similar to those of the control group.

### 2.3. Serum Serotonin Concentrations

**Figure 7:**
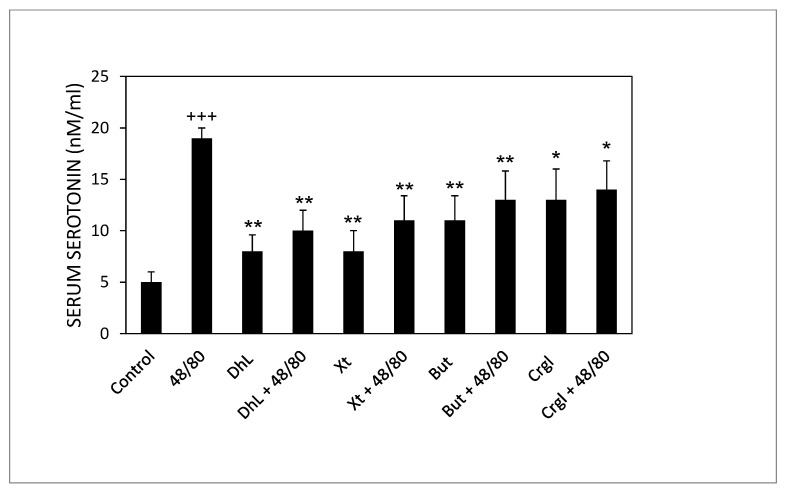
Effect of DhL, Xt, But, and Crgl on rat serum serotonin levels induced by compound 48/80. Values of serotonin concentrations are expressed as mean ± SEM +++ *p* < 0.001 when compared with the control group, ** *p* < 0.01 when compared with the compound 48/80 group, and * *p* < 0.05 when compared with the compound 48/80 group.

Rats treated with compound 48/80 alone had higher serum serotonin concentrations than control rats. Rats administered with the lactones (DhL, Xt, But, or Crgl) plus compound 48/80 showed lower levels of serum serotonin than rats treated with compound 48/80 alone (Figure 7).

## 3. Discussion

There are different research and review papers based on antiulcer screening models to determine the antiulcer activity of new drug molecules. Based on the methodology, antiulcer models can be classified as in vitro or in vivo. The animal is not required for in vitro models, while for in vivo models, animals are required to develop gastric lesions to determine the activity of a newly synthesized drug or molecule. The term in vivo directly indicates the evaluation of drugs in a living organism, and evaluation of antiulcer drugs is incomplete without using an in vivo model because the gastric ulcer is a disease that occurs in the body’s internal environment and forms lesions in humans. In all in vivo models, the examination of the gastric inner surface and calculation of the ulcer index are used as validated tools for assessing antiulcer activity [28,29,30].

The first relevant result of this work was the successful development of an experimental model of gastric ulcer mediated by mast cell activation. Our findings strongly indicate that the repeated administration of compound 48/80 results in extensive mucosal lesions in the rat gastric mucosa. We also showed that such lesions occurred in association with mast cell degranulation and with a significant increase of serum serotonin which may be responsible for the development of inflammatory lesions in the stomach.

Compound 48/80 is known to cause degranulation of connective tissue mast cells, such as peritoneal mast cells, with the release of serotonin and histamine from the cells [31,32,33,34,35,36,37,38]. Ohta et al. have shown that a single compound 48/80 treatment induces the release of endogenous biogenic amines through degranulation of connective tissue mast cells and the subsequent development of gastric mucosal lesions in male Wistar rats [39]. Yasuhiro et al. reported that in unanesthetized Sprague-Dawley rats with repeated compound 48/80 treatment, gastric mucosal lesions are caused primarily by the release of serotonin, but not histamine [40]. The ulcerogenic effect of serotonin on the stomach of experimental animals has been reported by many investigators [12,39]. In our laboratory, we failed to achieve the development of gastric ulcers through a single administration of compound 48/80. However, in the present study, we have shown that 4 days of treatment with compound 48/80, similar to that described by Yasuhiro et al. [40], caused gastric lesions mediated by endogenous serotonin. Histological changes support these results. Inflammatory lesions in the rat stomachs were accompanied by degranulation of mast cells in the gastric submucosa and a significant increase in serum serotonin. We interpret the discrepancy between our results and those obtained by Ohta et al. [39] to be due to the rat strain used.

We further showed that such lesions, induced by repeated compound 48/80 treatment, were prevented by pre-treatment with the optimal gastric cytoprotective dose (40 mg/kg of body weight) of dehydroleucodine, xanthatin, and 3-benzyloxymethyl-5*H*-furan-2-one, as expected. Furthermore, the effect obtained with each of these lactones was similar to that obtained with the lactone sodium cromoglycate, a reference mast cell stabilizer. In previous works, we have demonstrated that the potency of dehydroleucodine, xanthatin, and 3-benzyloxymethyl-5*H*-furan-2-one was higher than that of ketotifen and sodium cromoglycate to inhibit mast cell serotonin release from rat peritoneal mast cells induced by compound 48/80 [25,26]. In recent work, we have also shown that the inhibitory potency of dehydroleucodine and xanthatin was higher than that obtained with the reference compounds ketotifen and sodium cromoglycate when mast cells were preincubated with dehydroleucodine before substance P incubation and with dehydroleucodine or xanthatin before neurotensin incubation [27]. The administration of these lactones after compound 48/80 treatment had no effect on gastric lesions induced by the mast cell secretagogue (data not shown).

It is known that dehydroleucodine, xanthatin, and 3-benzyloxymethyl-5*H*-furan-2-one exert their mast cell stabilizer effect by inhibiting the degranulation and serotonin release from murine and human mast cells and that these lactones seem to block signaling pathways downstream of cytosolic calcium increase [25,26,27]. Moreover, we have also shown that dehydroleucodine, xanthatin, and 3-benzyloxymethyl-5*H*-furan-2-one prevent, in vivo and in a dose–response manner, the formation of gastrointestinal ulcers developed in response to ulcer-inducing agents, such as absolute ethanol [21,22]. These results suggest that the gastrointestinal cytoprotective and anti-inflammatory properties of dehydroleucodine, xanthatin, and 3-benzyloxymethyl-5*H*-furan-2-one may be explained by inhibition of mast cell activation. However, further research is needed in order to explain the exact molecular mechanisms of these actions.

In conclusion, the results of the present study indicate for the first time that the optimal gastric cytoprotective dose (40 mg/kg of body weight) of dehydroleucodine, xanthatin, and 3-benzyloxymethyl-5*H*-furan-2-one is efficacious in an animal model of gastric ulcer induced by mast cell activation. This study has some limitations, such as the difficulty of extrapolating results from animals to humans, the lack of data to establish the compounds’ pharmacological potency, and the problem of not being able to elucidate a mechanism of action for the drugs tested at the molecular level. Despite these limitations, our findings suggest that these lactones could be effective in treating peptic ulcer disease in humans and may become valuable tools for designing and developing novel therapeutic agents for digestive disorders associated with inappropriate mast cell activation.

## 4. Materials and Methods

### 4.1. Chemicals and Reagents

Compound 48/80, carboxymethylcellulose, sodium cromoglycate (Crgl), formaldehyde, hematoxylin, eosin, safranin, and toluidine blue were purchased from Sigma-Aldrich (St. Louis, MO, USA). Dehydroleucodine (DhL), xanthatin (Xt), and 3-benzyloxymethyl-5*H*-furan-2-one (But) were obtained as referred below. All other substances were supplied by Merck (Darmstadt, Germany). All chemicals used in these studies were of the highest grade available.

### 4.2. Animals

Male Sprague-Dawley adult rats (*n* = 100) weighing approximately 400 g, infection-free, and maintained under a 12-h dark/light cycle in a temperature-controlled room (24–25 °C) with free access to drinking water and laboratory food, were used for the experiments. All animals were cared for in accordance with the Guide for the Care and Use of Laboratory Animals of the US National Institutes of Health. All procedures were approved by the Institutional Animal Care and Use Committee of the School of Medical Sciences, Universidad Nacional de Cuyo (Protocol No. 182/2020).

### 4.3. Isolation and Purification of Dehydroleucodine

*Artemisia douglasiana* Besser was collected in the mountains of the province of San Luis, Argentina, in March 2019 and was identified by Prof. Luis A. Del Vitto. A voucher specimen was deposited in the Herbarium of the Universidad Nacional de San Luis (voucher 55-UNSL). Dehydroleucodine was isolated as previously described [25].

### 4.4. Isolation and Purification of Xanthatin

*Xanthium cavanillesii* Schouw was collected in El Volcán, Dpto. La Capital, San Luis, Argentina, in March 2019, and was identified by Prof. Luis A. Del Vitto. A voucher sample is available at the Herbarium of the Universidad Nacional de San Luis (voucher 8985-UNSL). Xanthatin was isolated as previously described [25].

### 4.5. Preparation of 3-Benzyloxymethyl-5H-furan-2-one

3-Benzyloxymethyl-5*H*-furan-2-one was prepared as previously described [25].

### 4.6. General Protocol and Induction of Gastric Ulcer by Mast Cell Activation

**Figure 8:**
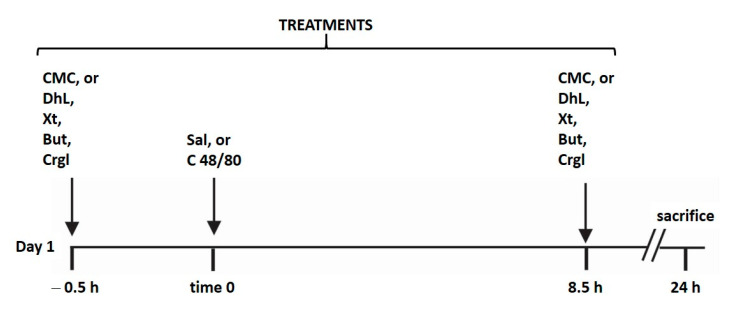
Schematic representation illustrating the experimental design of the study. The animals (*n* = 100) were randomly divided into ten groups (*n* = 10 in each group). Each group was treated as detailed in the scheme. Treatments of all groups were repeated for four days. CMC: 0.4% carboxy-methylcellulose; DhL: 40 mg/kg BW dehydroleucodine; Xt: 40 mg/kg BW xanthatin; But: 40 mg/kg BW 3-Benzyloxymethyl-5*H*-furan-2-one; Crgl: 40 mg/kg BW sodium cromoglycate; Sal: saline solution; C 48/8: 0.75 mg/kg BW compound 48/80.

All rats for the experiments were housed in wire-mesh-bottom cages throughout the study to prevent coprophagy. All animals were maintained with free access to water and without food during the experiment. Gastric lesions were produced according to the method of Yasuhiro et al. [40] with some modifications performed by us (Figure 8). The animals were randomly divided into ten groups (*n* = 10 in each group):

Group I (control): rats were treated with 0.4% carboxy-methylcellulose (CMC; 0.10 mL/10 g BW), administered intragastrically (i.g.), 30 min before and 8.5 h after the intraperitoneal (i.p.) injection of saline solution.

Group II (48/80): rats were treated with 0.4% carboxy-methylcellulose (CMC; 0.10 mL/10 g BW), administered intragastrically (i.g.), 30 min before and 8.5 h after the intraperitoneal (i.p.) injection of 0.75 mg/kg BW compound 48/80.

Group III (DhL): rats were treated with DhL suspended in 0.4% CMC at a dose of 40 mg/kg BW, administered intragastrically (i.g.), 30 min before and 8.5 h after the intraperitoneal (i.p.) injection of saline solution.

Group IV (DhL + 48/80): rats were treated with DhL suspended in 0.4% CMC at a dose of 40 mg/kg BW, administered intragastrically (i.g.), 30 min before and 8.5 h after the intraperitoneal (i.p.) injection of 0.75 mg/kg BW compound 48/80.

Group V (Xt): rats were treated with Xt suspended in 0.4% CMC at a dose of 40 mg/kg BW, administered intragastrically (i.g.), 30 min before and 8.5 h after the intraperitoneal (i.p.) injection of saline solution.

Group VI (Xt + 48/80): rats were treated with Xt suspended in 0.4% CMC at a dose of 40 mg/kg BW, administered intragastrically (i.g.), 30 min before and 8.5 h after the intraperitoneal (i.p.) injection of 0.75 mg/kg BW compound 48/80.

Group VII (But): rats were treated with But suspended in 0.4% CMC at a dose of 40 mg/kg BW, administered intragastrically (i.g.), 30 min before and 8.5 h after the intraperitoneal (i.p.) injection of saline solution.

Group VIII (But + 48/80): rats were treated with But suspended in 0.4% CMC at a dose of 40 mg/kg BW, administered intragastrically (i.g.), 30 min before and 8.5 h after the intraperitoneal (i.p.) injection of 0.75 mg/kg BW compound 48/80.

Group IX (Crgl): rats were treated with Crgl suspended in 0.4% CMC at a dose of 40 mg/kg BW, administered intragastrically (i.g.), 30 min before and 8.5 h after the intraperitoneal (i.p.) injection of saline solution.

Group X (Crgl + 48/80): rats were treated with Crgl suspended in 0.4% CMC at a dose of 40 mg/kg BW, administered intragastrically (i.g.), 30 min before and 8.5 h after the intraperitoneal (i.p.) injection of 0.75 mg/kg BW compound 48/80.

Treatments of all groups were repeated for four days. All the experiments were performed simultaneously. Animals were sacrificed by CO_2_ inhalation 24 h after the last administration of saline solution or compound 48/80. Their stomachs were removed, opened along the greater curvature, and washed gently with ice-cold saline solution. Immediately after the collection, the degree of erosion was assessed with a scoring system and the observation of histological preparations for light microscopy. Gastric mast cell morphology was analyzed in all samples by histological procedures with special stains. Serum serotonin levels were determined as markers of mast cell activation.

### 4.7. Scoring System

The degree of erosion was assessed from a scoring system designed by Marazzi-Uberti and Turba [41] as follows: 0, no erosions; 1, 1–3 small erosions (4 mm diameter or smaller); 2, more than 3 small erosions or one large erosion; 3, one large erosion and more than 3 small erosions; 4, 3–4 large erosions; 5, any very large erosion or ulcer perforation. The results were expressed in terms of an ulcer factor, which is the average severity of erosions *per* rat for each group on a scale from 0 to 5. The sum of these values was divided by the number of animals. This procedure was performed by two observers using a Nikon binocular stereomicroscope (×40 magnification).

### 4.8. Light Microscopy

The samples for light microscopy were fixed for 24 h in a 10% formaldehyde solution (prepared with saline solution, pH = 7), dehydrated in graded ethanol and xylol, and embedded in paraffin. Serial sections (6 µm) were mounted on glass slides and deparaffinized. Some sections were stained with hematoxylin-eosin in order to evaluate the general histoarchitecture and the degree of gastric lesions. Other sections were stained with 1% toluidine blue and with 0.5% safranin in order to reveal mast cell granules [42].

### 4.9. Determination of Serum Serotonin

For serum serotonin determinations, blood was collected from the inferior vena cava of rats upon sacrifice. Then, serum was obtained from the collected blood by centrifugation. Serum samples were deproteinized by adding perchloric acid at a final concentration of 3% and then centrifuged (1000× *g*, 4 °C, 10 min). Serum serotonin was measured by high-performance liquid chromatography (HPLC) with electrochemical detection, as previously described [27].

### 4.10. Statistics

Multiple comparisons of the difference of independent treatment groups were made by ANOVA-1 design and the Tukey–Kramer test, where Control, Compound 48/80, DhL, DhL + 48/80, Xt, Xt + 48/80, But, But + 48/80, Crgl and Crgl + 48/80 were treatments, such as is shown in Figure 8. A probability of *p* < 0.05 was considered statistically significant.
